# Identification of Three Significant Genes Associated with Immune Cells Infiltration in Dysfunctional Adipose Tissue-Induced Insulin-Resistance of Obese Patients via Comprehensive Bioinformatics Analysis

**DOI:** 10.1155/2021/8820089

**Published:** 2021-01-22

**Authors:** Ming Zhai, Peipei Luan, Yefei Shi, Bo Li, Jianhua Kang, Fan Hu, Mingjie Li, Lei Du, Donglei Zhou, Weixia Jian, Wenhui Peng

**Affiliations:** ^1^Department of Cardiology, Shanghai Tenth People's Hospital, School of Medicine, Tongji University, 301 Middle Yanchang Road, Shanghai 200072, China; ^2^Department of Endocrinology, Xinhua Hospital, Shanghai Jiaotong University, School of Medicine, 1665 Kongjiang Road, Shanghai 200092, China; ^3^Department of Endocrinology and Metabolism, Shanghai Jiao Tong University Affiliated Sixth People's Hospital, 600 Yishan Road, Shanghai 200233, China; ^4^Department of Metabolic Surgery, Shanghai Tenth People's Hospital, School of Medicine, Tongji University, 301 Middle Yanchang Road, Shanghai 200072, China; ^5^Department of General Surgery, Shanghai Tenth People's Hospital, School of Medicine, Tongji University, 301 Middle Yanchang Road, Shanghai 200072, China

## Abstract

**Background:**

Low-grade chronic inflammation in dysfunctional adipose tissue links obesity with insulin resistance through the activation of tissue-infiltrating immune cells. Numerous studies have reported on the pathogenesis of insulin-resistance. However, few studies focused on genes from genomic database. In this study, we would like to explore the correlation of genes and immune cells infiltration in adipose tissue via comprehensive bioinformatics analyses and experimental validation in mice and human adipose tissue.

**Methods:**

Gene Expression Omnibus (GEO) datasets (GSE27951, GSE55200, and GSE26637) of insulin-resistant individuals or type 2 diabetes patients and normal controls were downloaded to get differently expressed genes (DEGs), and GO and KEGG pathway analyses were performed. Subsequently, we integrated DEGs from three datasets and constructed commonly expressed DEGs' PPI net-works across datasets. Center regulating module of DEGs and hub genes were screened through MCODE and cytoHubba in Cytoscape. Three most significant hub genes were further analyzed by GSEA analysis. Moreover, we verified the predicted hub genes by performing RT qPCR analysis in animals and human samples. Besides, the relative fraction of 22 immune cell types in adipose tissue was detected by using the deconvolution algorithm of CIBERSORT (Cell Type Identification by Estimating Relative Subsets of RNA Transcripts). Furthermore, based on the significantly changed types of immune cells, we performed correlation analysis between hub genes and immune cells. And, we performed immunohistochemistry and immunofluorescence analysis to verify that the hub genes were associated with adipose tissue macrophages (ATM).

**Results:**

Thirty DEGs were commonly expressed across three datasets, most of which were upregulated. DEGs mainly participated in the process of multiple immune cells' infiltration. In protein-protein interaction network, we identified *CSF1R*, *C1QC*, and *TYROBP* as hub genes. GSEA analysis suggested high expression of the three hub genes was correlated with immune cells functional pathway's activation. Immune cell infiltration and correlation analysis revealed that there were significant positive correlations between *TYROBP* and M0 macrophages, *CSF1R* and M0 macrophages, Plasma cells, and CD8 T cells. Finally, hub genes were associated with ATMs infiltration by experimental verification.

**Conclusions:**

This article revealed that *CSF1R*, *C1QC*, and *TYROBP* were potential hub genes associated with immune cells' infiltration and the function of proinflammation, especially adipose tissue macrophages, in the progression of obesity-induced diabetes or insulin-resistance.

## 1. Introduction

Recently, obesity became one of the major health concerns as it contributed to the growing prevalence of its related comorbidities, including insulin resistance (IR) and type 2 diabetes. “Diabesity” is a new term which refers to diabetes or IR occurring in the context of obesity [1].

According to a report from International Diabetes Federation, overweight and obesity account for 65% to 80% of the increase in the prevalence of type 2 diabetes. Growing evidences show that chronic inflammation in adipose tissue is a key factor in the development of IR and type 2 diabetes in obese individuals [2, 3]. However, the pathogenesis underlying obesity and IR has not yet been fully clarified. Recently, more and more researches supported that the chronic inflammation of obese adipose tissue plays a crucial role in the progression of IR in diabetes patients [4–6]. While classically, the chronic inflammation has been identified by increased cytokines or chemokines expression, such as upregulated TNF-*α*, IL-6, and interferon (IFN) *γ*, immune cell infiltration is a prominent feature of dysfunctional adipose tissue (AT), as well. These immune cells include M1 and M2 polarization macrophages, effector and memory T cells, FoxP3^+^T regulatory cells, natural killer (NK), and NKT cells [7]. Infiltrating immune cells secrete not only proinflammation cytokines but also metalloproteinases and chemokines, which participates in immune cell signaling and regulation of immunity in dysfunctional AT [8]. Dysfunctional AT also releases adipokines such as leptin, resistin, and visfatin which break the systemic homeostasis, alter the glucose metabolism, and lead to insulin resistance [9]. Therefore, figuring out how the immune cell infiltration and inflammation-related critical genes' function in these immune cells is important to clarify the potential mechanisms.

Bioinformatic analysis has been widely used in sequencing, microarray gene expression analysis, single-cell sequencing, and human microbiota [10, 11]. Integrated bioinformatics analysis enables researchers to quickly identify differentially expressed target genes and their related functional pathways, which can serve as a valuable guide for further exploration [12–16]. Although immune cell infiltration in the adipose tissue played a critical role in diabetes, few studies were designed to explore the relationship between immune cells infiltration and related hub genes' pathogenetic function in adipose tissue by the utility of datasets in National Center Biotechnology Information Gene Expression Omnibus (GEO).

Several studies show that obesity changes the number and function of the various types of immune cells in the AT [4, 17]. As mentioned before, AT chronic inflammation induces AT dysfunction and further leads to metabolic imbalance or IR/diabetes. So, it is necessary to understand the molecular mechanism that underlies the development of obesity-induced IR. In our present study, the microarray data of three datasets were downloaded from the GEO website. Each dataset contains transcriptome data of adipose tissue of IR or diabetic patients under obesity condition as case group and adipose tissue of healthy and lean people as control group. Therefore, these datasets provide us an opportunity to mining valuable regulating hub genes participating in the process of dysfunctional adipose immune cells' infiltration, which will link obesity-induced autoinflammation to IR or diabetic pathogenesis. By using limma package in R studio, we screened commonly differentially expressed genes (DEGs) across three datasets. Next, the package “ClusterProfiler” was used for DEGs' functional annotation in three datasets, respectively, to judge whether the DEGs were related to proinflammation function or pathway. After verification, we established protein-protein interaction (PPI) network, and then, we applied MCODE (Molecular Complex Detection) and CytoHubba plugin in Cytoscape software to find the hub genes regulating the DEGs community relative to proinflammation. Then, the GSEA analysis was carried out based on our downloaded datasets to find out the most significant functional terms participating in the hub gene regulation progress. By using CIBERSORT, we first investigated the differences in 22 subpopulations of immune cells' infiltration within AT between IR combined obese group and control group; furthermore, the graph of relationship between 22 subpopulations of immune cells and screened hub genes was constructed and then verified by experiments. Screened hub genes were proven to be upregulated in AT accompanied with the process of obtained obesity-induced IR. Our aim is to find significant hub genes associated with immune cell infiltration in dysfunctional AT-induced IR that affect clinical manifestation and to provide a new direction for investigating the pathological mechanism of chronic inflammation-induced metabolism imbalance.

## 2. Materials and Methods

### 2.1. Obese Patients with Diabetes or IR and Controls

This study was approved by the Ethics Committee of Shanghai Tenth People's Hospital. The written informed consent was collected from each patient or their relatives. Five obese patients diagnosed with type 2 diabetes or IR (IRO patients) were selected for our research. These patients underwent bariatric surgery at Shanghai Tenth People's Hospital from May to June in 2020, and the visceral adipose tissue (VAT) (came from omental adipose tissue) and subcutaneous adipose tissue (SAT) samples were collected after obtaining informed consents. Meanwhile, we also recruited 5 lean patients without diabetes or IR who underwent laparoscopic surgery, and the VAT or SAT samples were also collected after obtaining the patients' agreements, which served as control groups.

The inclusion criteria of IRO group were as follows: (1) diagnosis of type 2 diabetes; (2) BMI index over 30 kg/m^2^; and (3) no cancer-related diagnosis. And, the inclusion criteria of control group were as follows: (1) No type2 diabetes or IR; (2) BMI index less than 24 kg/m^2^; and (3) no cancer-related diagnosis. The clinical data on medical histories, height, weight, HbA1c, and other data were summarized in Supplemental Table 1. Fasting plasma glucose was measured with standard laboratory techniques on a Hitachi 7104 Analyzer (Hitachi, Tokyo, Japan). HbA1c was determined by HPLC (Hi-Auto HA-8150; Arkray, Kyoto, Japan).

### 2.2. Animal Study

Animal studies were conducted according to the Guide for the Care and Use of Laboratory Animals (U.S. National Institutes of Health (NIH) Bethesda, MD, USA), and all procedures or methods were approved by the Animal Care and Use Committee of Tenth people's hospital affiliated of Tongji University School of Medicine [18]. 6-week-old male C57BL/6 mice (20 g) were purchased from Shanghai SLAC Laboratory Animal Co., Ltd and adapted for 2 weeks in raising room. Animals were housed in cages of raising room with a cycle of 12 : 12 h light/dark and a suitable temperature (22 °C–25 °C). At 8 weeks of age, 14 male mice were randomly divided into 2 groups and fed for 12 weeks as follows: normal diet (ND, *n* = 7) feeding and high-fat diet feeding (HFD, 60% of calories from fat, research diets, D12492, *n* = 7). The food intake and body weight of each mouse were recorded weekly. After 12 weeks, we performed glucose tolerance tests of each mouse. Blood glucose concentrations (mg/dl) were measured following fasting, prior to the test, and 15, 30, 60, and 120 minutes after intraperitoneal injection of 20% glucose (2 mg/g body weight) (SIGMA). Then, blood glucose levels were determined from tail vein blood using an automatic glucometer (Bayer Contour, Bayer, Germany). After 12 weeks, we collected VAT (Epididymis adipose tissue) and SAT from 2 groups, respectively, for further analysis.

### 2.3. Microarray Datasets

The National Center Biotechnology Information Gene Expression Omnibus (https://http://www.ncbi.nlm.nih.gov/geo/) website was regarded as our data resources [19]. We searched these datasets based on following standards: (i) adipose tissue of IRO; (ii) adipose tissue of lean insulin-sensitive (IS) people without diabetes as control. The datasets of GSE27951, GSE55200, and GSE26637 were selected. The platform for GSE27951 was GPL570 [HG-U133_Plus_2] Affymetrix Human Genome U133 Plus 2.0 Array, and we chose 5 samples *(GSM691143*, *GSM691147*, *GSM691150*, *GSM691153*, and *GSM691154)* of adipose tissues obtained from lean and healthy people and 5 samples *(GSM691125*, *GSM691129*, *GSM691138*, *GSM691142*, and *GSM691144)* of adipose tissues obtained from diabetic and obese people from the datasets. The platform for GSE55200 was GPL17692 [HuGene-2_1-st] Affymetrix Human Gene 2.1 ST Array, and we selected 7 samples *(GSM1331431*, *GSM1331432*, *GSM1331433*, *GSM1331434*, *GSM1331435*, *GSM1331436*, and *GSM1331437)* of adipose tissues from lean and healthy people and 8 samples (*GSM1331446*, *GSM1331447*, *GSM1331448*, *GSM1331449*, *GSM1331450*, *GSM1331451*, *GSM1331452*, and *GSM1331453)* of adipose tissues from metabolic unhealthy or insulin-resistant obese people. The platform for GSE26637 was also GPL570 [HG-U133_Plus_2] Affymetrix Human Genome U133 Plus 2.0 Array, and we chose 10 samples *(GSM655608*, *GSM655609*, *GSM655610*, *GSM655611*, *GSM655612*, *GSM655618*, *GSM655619*, *GSM655620*, *GSM655621*, and *GSM655622)* of subcutaneous adipose tissues from lean and IS people and 9 samples *(GSM655603*, *GSM655604*, *GSM655605*, *GSM655607*, *GSM655613*, *GSM655614*, *GSM655615*, *GSM655616*, and *GSM655617)* of adipose tissues from obese and insulin-resistant people. Series matrix file(s) were downloaded. Furthermore, to better verify the correction between hub genes and the process of obesity induced diabetes, we introduced a GSE35411; the platform of it is GPL10335 Affymetrix Human Genome U133 Plus 2.0 Array, which contained expression changes of hub genes from different clinical status.

And, the clinical data were obtained from the related published paper [20]. Series matrix file(s) and GPL-annotated files were downloaded.

### 2.4. Analysis of Differentially Expressed Genes

The “limma” package in R software (R Studio) was used to integrate and standardize the downloaded GEO expression chip to analyze the differentially expressed genes and their expression levels [21, 22]. The differentially expressed genes were screened under the condition of |log FC|(log FC) >1 and adjusted *P* value <0.05. The differential gene heatmap was drawn using the R package “PheatMap” package. All statistical analyses were performed in the R language (Version 3.6). All statistical tests were bilateral, and adjusted *P* value <0.05 was statistically significant. And, the common differentially expressed genes across three datasets were performed by the Venn diagram package in R software.

### 2.5. DEGs Pathway Enrichment Analyses

We used the R package “ClusterProfiler” for functional annotation of differential genes to fully explore the functional correlation of DEGs from three datasets, respectively [23]. Genetic ontology (GO) and the Kyoto Encyclopedia of Genes and Genomics (KEGG) were used to assess the relevant functional categories [24, 25]. GO and KEGG enrichment pathways with adjust *P* value less than 0.05 were considered as significant categories.

### 2.6. Protein-Protein Interaction Network Analysis

Protein-protein interaction (PPI) information could be obtained by an online tool, Search Tool for the Retrieval Interacting Genes (STRING) [26]. Cytoscape 3.6.0 was an open-access tool for visualizing the network of genes and proteins. PPI of the DEGs was evaluated from the STRING database and was constructed by Cytoscape [27]. In addition, the Molecular Complex Detection (MCODE; version 1.31) is a plugin of Cytoscape, used to analyze modules of the PPI network (degree cutoff = 2, max. depth = 100, *k*-core = 2, and node score cutoff = 0.2). Furthermore, we used the CytoHubba, a plugin of Cytoscape, to screen hub genes through 3 terms of degrees, closeness, and betweenness in the module visualized by the MCODE.

### 2.7. Gene Set Enrichment Analysis

Gene Set Enrichment Analysis (GSEA, version 4.1.0, the broad institute of MIT and Harvard) was used to discuss whether a genetically defined genome is statistically significant between the two groups of samples [18]. GSEA was performed to identify activated Reactome or KEGG gene sets pathways in the groups of high expression of three hub genes, respectively, where we considered FDR *q* value <0.05.

### 2.8. Immune Cell Infiltration Analysis and Immune Cell Correlation Analysis

First, we downloaded Series Matrix File of GSE55200 from NCBI GEO public database, with 15 samples included. GPL annotation platform was GPL17692; GSE84599 Series Matrix File including a total of 16 transcriptome data was also downloaded, and the GPL annotation platform was GPL16699. The CIBERSORT algorithm, a deconvolution computational method for quantifying immune cell fractions from tissue gene expression profiles [28, 29], was used to analyze transcriptome data of GSE55200 patients from different subgroups to infer the relative proportion of immune-infiltrating cells. The “Vioplot” package was used to plot the relative content of immune cells. To evaluate the influence of genes on immune infiltration, CIBERSORT algorithm was used to quantify the infiltration level of immune cells in each sample of GSE84599, and then spearman correlation analysis was conducted for specific gene expression and immune cell content. The statistical analysis was conducted in R language (Version 3.6). All statistical tests were bilateral. *P* value <0.05 was considered statistically significantly.

### 2.9. RNA Isolation and Real-Time qPCR

Total RNA from adipose tissue was extracted with Trizol reagent (Thermo Fisher Scientific). Reverse transcription was performed using 1 ug of RNA and HiScript III RT SuperMix reverse-transcription reagent kit (Vazyme Biotech Co., Ltd, Nanjing, China). Real-time qPCR was performed on a LightCycler 96 Real-Time PCR System (F. Hoffmann-La Roche) using FastStart Essential DNAGreen Master assay (F. Hoffmann-LaRoche). The 2^−ΔΔCt^ method was used for semiquantitative analysis. 18s rRNA and 36B4 served as internal standard. Primers are listed in Supplemental Table 2.

### 2.10. Histology and Immunohistochemistry (IHC) Test

Adipose tissues were fixed in 10% formalin, processed, and paraffin embedded for sectioning into 3-mm thick sections. Immunohistochemical tests for CSF1R (sc-46662; Santa Cruz Biotechnology, USA), C1QC (ab75756; Abcam, UK), and CD68 (NBP233337, Novus Biologicals, USA) were performed on paraffin-embedded sections. Microwave-based antigen retrieval was performed using 10 mM/L sodium citrate buffer. Sections were then incubated with 0.3% hydrogen peroxide diluted with methanol for 15 min to inactivate endogenous peroxidase activity. After 3 washes in PBS and being blocked in 10% goat serum for 30 min at room temperature, sections were incubated with primary antibody overnight at 4°C. A standard ABC-peroxidase system (Vector Laboratories, Burlingame, CA, USA) was used to detect primary antibodies. Positive antibody binding was visualized using a DAB peroxidase substrate kit (SK-4100; Vector Laboratories). Image-J version 1.53 (National Institutes of Health, USA) software was used for quantizing the positive area of three hub genes, respectively.

### 2.11. Immunofluorescence (IF) Analysis

Fresh human omental visceral adipose tissues were embedded in optimal cutting temperature compound (4583; Sakura Finetek Japan, Tokyo, Japan) and sliced into 5-mm sections. After the frozen sections rewarming, the sections were washed 3 times with PBS, fixed with acetone, and permeabilized with 0.2% Triton X-100 at room temperature for 10 min. After blocking for 30 min with 3% bovine serum albumin, the sections were incubated with anti-CSF1R (sc-4662; Santa Cruz), anti-CD68 (ab213363; Abcam) or anti-CD68(ab955), and anti-C1QC (ab75756; Abcam) antibodies for 3 h at room temperature. After washing with PBS, secondary antibodies (Alexa Fluor 488-conjugated goat anti-rabbit, Alexa Fluor 594-conjugated goat anti-mouse, Alexa Fluor 594-conjugated goat anti-rabbit, or Alexa Fluor 488-conjugated goat anti-mouse; Thermo Fisher Scientific) were incubated for 1 h at 37°C in the dark. Nuclei were labeled with DAPI, and sections were visualized and collected under the Nikon inverted fluorescence microscope (NIKON ECLIPSE TI-SR Japan).

### 2.12. Statistical Analysis

For analysis of gene and protein expression in mouse and human, GraphPad Prism 8.0 (GraphPad Inc., San Diego, CA, USA) was used for statistical analysis. Differences of variances among groups were estimated by two-way ANOVA or 2-tailed Student's *t* test. Data were presented as the mean ± standard deviation (SD). *P* value <0.05 was considered to indicate a statistical significance.

## 3. Results

### 3.1. Bioinformatic Analysis Workflow

**Figure 1:**
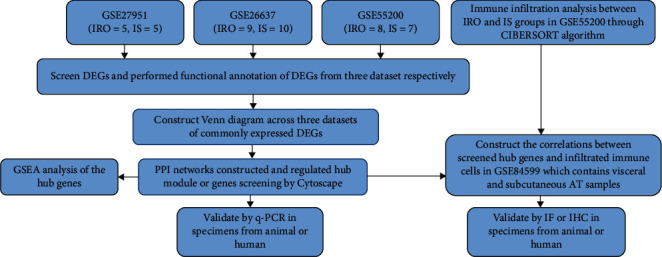
Workflow of our whole study design.

Our workflow is shown in Figure 1. We first integrated three datasets including transcriptome data of adipose tissue between IRO and IS group to screen each dataset's significant DEGs, and then the commonly regulating network across three datasets was constructed to find the potential functional critical modules and hub genes participating in the immune cell infiltration induced AT dysfunction, and GSEA analysis was performed to test what kinds of pathogenesis pathways that screened hub genes were highly correlated. At the same time, the immune cells infiltration analysis between IRO and IS group was carried out to find infiltrated differentially significantly immune cells in the dataset GSE55200 through CIBERSORT algorithm, and then we explored the correlation between three screened hub genes and immune cells, to find whether three screened hub genes involved in the process of abovementioned significantly immune cell's infiltration in dataset GSE84599 including more comprehensive samples from subcutaneous AT and visceral AT of IRO patients. Finally, the hub genes relative mRNA expression was verified in SAT or VAT from patients or animals modules; furthermore, the correlation between hub genes and infiltrated differently significantly immune cells with highest fraction in AT of IRO was verified by IF or IHC analysis.

### 3.2. Identification of DEG in IRO Groups vs. IS Groups

**Figure 2:**
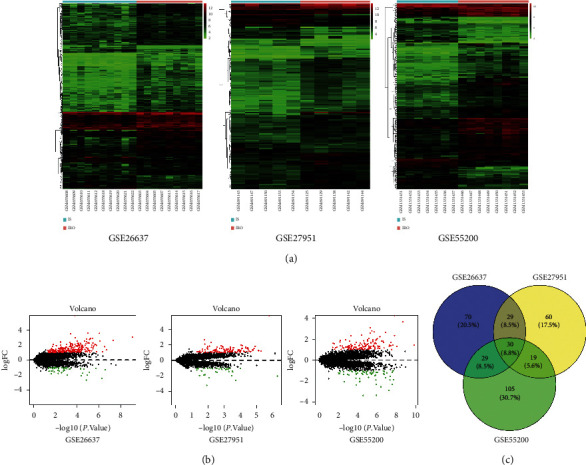
DEGs commonly expressed across three datasets. (a) The hierarchical clustering heatmap of upregulated and downregulated DEGs in the IRO group or IS group of each dataset. (b) The volcano plot shows upregulated genes (red points) and downregulated genes (green points) and genes without significance (black points). The differences threshold was set as |log2FC| >1.0 and adjusted *P* value <0.05. (c) The Venn diagram shows thirty DEGs commonly expressed across three datasets.

We used “limma” packages in R software to extract 138, 183, and 158 DEGs in the IRO/IS groups from GSE27951, GSE55200, and GSE26637, respectively. Then, we used the “VennDiagram” package in R software to screen the common DEGs among three datasets (Figure 2), and the adjust *P* value and logFC value of 30 common expressed DEGs are shown in the Supplemental Table 3. Results showed that a total of 30 commonly expressed DEGs across three datasets were detected.

### 3.3. Functional Annotation

**Figure 3:**
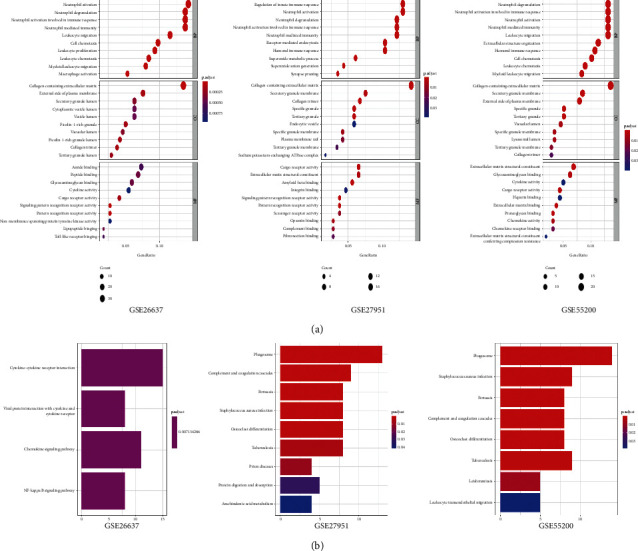
Functional annotation of DEGs from each dataset. (a) The cell component, molecular function, and biological process of DEGs annotated in gene ontology. Counts: the number of enriched DEGs. Gene Ratio: the number of expression genes in the GO category to that of the annotated genes. (b) The enriched KEGG pathways of DEGs from each dataset.

We uploaded DEGs from three datasets, respectively, to perform the GO analysis and KEGG analysis by using the “ClusterProfiler” package in R software. The GO biological process analysis found that DEGs were mainly enriched in innate immune response, extracellular and transmembrane cell receptors, and signaling transmission cell functions. Meanwhile, the performed KEGG pathway analysis revealed that the DEGs from three datasets were mainly enriched in inflammation caused by multiple inflammation cells, such as cytokine-interaction, phagosome, and complement cascades, as is shown in Figure 3.

### 3.4. PPI Analysis and Module Analysis

**Figure 4:**
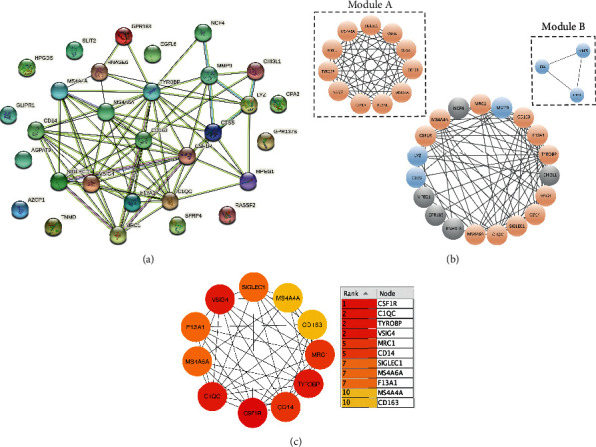
PPI networks and module analysis of commonly expressed DEGs across three datasets. (a) The PPI networks constructed by the STRINGs. (b) MCODE analysis of PPI networks. Module A score = 9.886; Module *B* = 3.0. (c) The major PPI network analyzing of top 11 hub genes through Cytohubba software. The shade of node's color reflects the degree of connectivity.

We imported 30 DEGs into PPI network complex, and a network including 30 nodes and 77 edges was constructed as depicted in Figure 4(a). We then used CytoHubba App to in Cytoscape to examined 30 DEGs through 3 terms of degrees, closeness, and betweenness, which is shown in the Supplemental Table 4. Next, Cytoscape MCODE software was used for further analysis, and the results constituted two modules: A and B, including 11 and 3 genes, respectively. According to the scores of two modules, the module A was considered as a critical module in the DEGs, which is shown in Figure 4(b). Then, we also used CytoHubba to screen hub genes through abovementioned 3 terms in the module A, and *CSF1R*, *C1QC*, and *TYROBP* were considered as hub genes in the module A, as is illustrated in Figure 4(c) and Supplemental Table 5.

### 3.5. GSEA Analysis

**Figure 5:**
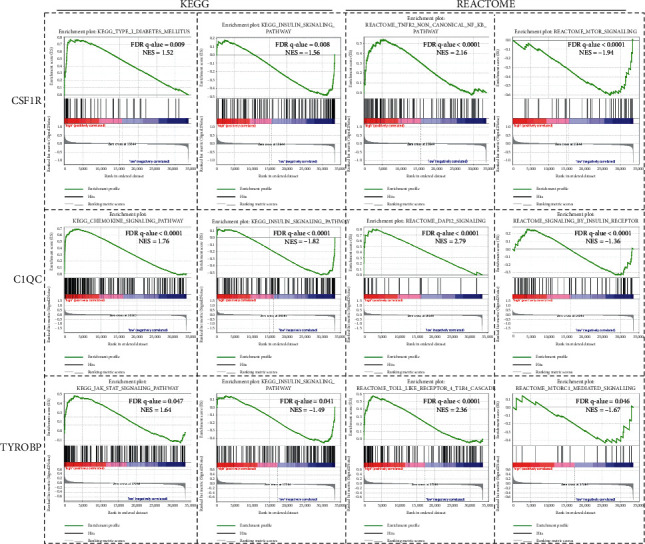
Gene set enrichment analysis between each hub gene's high and low expression samples. GSEA analyzing gene-expression signatures of datasets on the basis of each gene's high and low expression by using KEGG pathway gene sets and Reactome pathway gene sets.

Considering the GO analysis results that DEGs mainly enriched in inflammation response and cytokines response, we performed GSEA analysis of three hub genes in KEGG pathway and Reactome pathway, respectively. According to the median of each gene's expression value, we divided the samples from GSE55200 dataset into high-expression group and low-expression group, and then we performed GSEA between each hub gene's high- and low-expression group, as shown in Figure 5. Type 1 diabetes mellitus and NF-*κ*B pathway were activated in the *CSF1R* highly expressed group. As for the *C1QC* highly expressed group, Chemokine signaling and *DAP12* pathway were activated. Meanwhile, JAK-STAT signaling and TLR4 cascade pathway were activated in the *TYROBP* highly expressed group. On the contrary, insulin sensitivity-related insulin signaling and mTOR signaling pathway were significantly inhibited in three hub genes' highly expressed group.

### 3.6. Hub Genes' Upregulation in AT Was Accompanied with Process of Obtained Obesity-Induced IR

**Figure 6:**
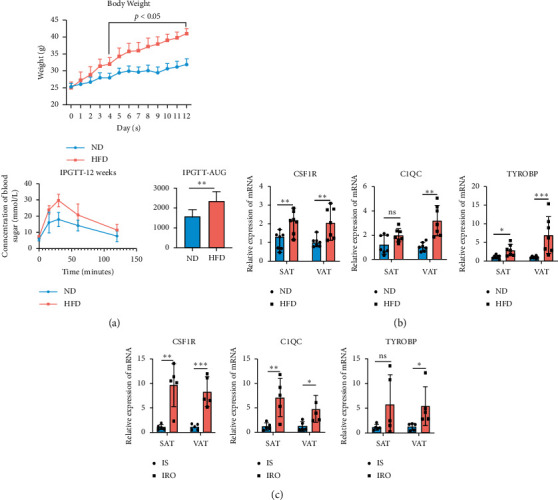
Hub genes' upregulation in AT was accompanied with process of obtained obesity-induced IR. Validation of *CSF1R*, *C1QC*, and *TYROBP* gene expressions levels by using RTqPCR. (a) Body weight curves of HFD feeding (*n* = 7) and ND feeding controls (*n* = 7) mice. Glucose tolerance tests (GTT) were performed in HFD fed mice and ND fed mice after 12 weeks feeding period. (b) mRNA expression levels of the top 3 hub genes in adipose tissue of SAT or VAT from HFD feeding mice and ND feeding control mice after 12 weeks (*n* = 7). (c) mRNA expression levels of the top 3 hub genes in adipose tissue of SAT or VAT from IRO patients and IS controls (*n* = 5). Unpaired two-sided *t* test, ^*∗*^*P* value <0.05. ^*∗∗*^*P* value <0.01. ^*∗∗∗*^*P* value <0.001. n.s. = not significant.

To verify the results of top 3 hub genes predicted by bioinformatic analysis, we performed Rt-qPCR analysis in SAT or VAT tissues from HFD-induced obese mice and obese diabetic patients as well. The body weight curves in the feeding period of 12 weeks and IPGTT curves after 12 weeks feeding are shown in Figure 6(a). The body weight curve reflected the HFD-fed mice gained significant weight compared with ND-fed mice since 4^th^ week; meanwhile, the IPGTT curve showed that, at the end of feeding period of 12 weeks, the HFD-fed group mice were presented with drastically IR compared with the ND-fed group mice. After 12 weeks feeding, the hub genes' expressions were verified in the SAT or VAT tissue isolated from ND groups and HFD groups, respectively. The RTqPCR results showed that three hub genes were upregulated in SAT or VAT tissues from HFD groups, which are shown in Figure 6(b). Meanwhile, RT-qPCR results in the SAT tissues and VAT tissues from IRO groups and IS groups showed that the upregulated trends of hub genes were consistent with that in HFD-induced obese mice groups, which is shown in Figure 6(c). Furthermore, to verify the correlation between hub genes and the process of obesity induced diabetes, we introduced a GSE35411 including correlating transcriptomic data of 18 AT samples obtained from 9 patients' AT within 2 clinical phase, respectively, including baseline (before weight loss) phase and weight maintenance phase (after diet control and exercise). We compared the expression of hub genes *CSF1R*, *C1QC*, and *TYROBP*, respectively, between the two clinical phases. And, we found that the hub genes were all downregulated along with the weight losing and IS improving in weight-maintenance phase, as is shown in Supplemental Figure 1.

### 3.7. Immune Cell Infiltration Analysis and Immune Cell Correlation Analysis

**Figure 7:**
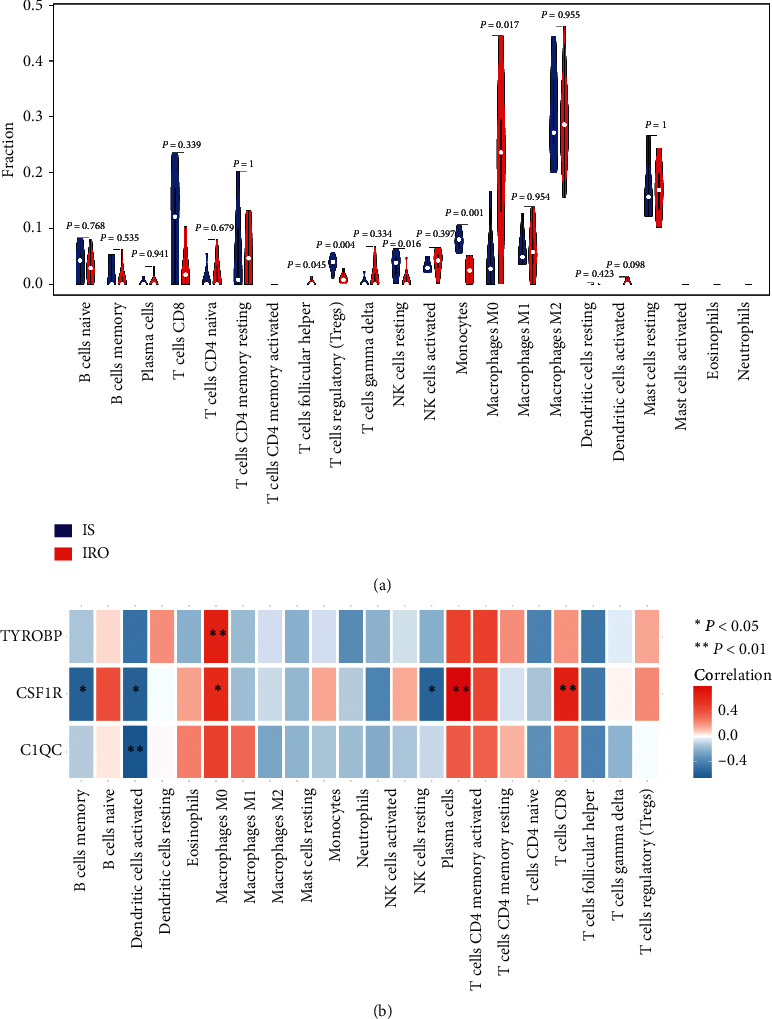
Immune cell infiltration analysis and correlation analysis. (a) Significant changes in infiltrated immune cells in IRO compared with IS groups (Wilcoxon test *P* value <0.05). (b) Correlation between gene expressions and the relative percentages of immune cells in the inflammatory adipose tissue.

We performed the immune cell infiltration analysis by using the CIBERSORT algorithm to investigate the general proportion of the 22 subpopulations of immune cells among samples from IRO and IS groups, respectively, in GSE55200. We found that 5 types of immune cells were significantly different between the IS and IRO groups (Figure 7(a)). The cells types were T cells follicular helper, T cells regulatory, NK cells resting, monocytes, and macrophages M0. Among the immune cells, we found that T cells follicular helper and macrophage M0 in the IRO group were presented at higher fractions than in the IS group, and the M0 macrophage shows the highest fraction within them in AT of IRO group, while the other 3 types of immune cells showed the opposite results. Furthermore, we performed the correlation analysis between the 22 kinds of immune cells and hub genes by using GSE84599. As shown in the Figure 7(b), *TYROBP* and M0 macrophages, *CSF1R* and M0 macrophages, Plasma cells, or CD8 T cells showed significant positive correlations.

### 3.8. Increased Expression of Hub Genes Is Consistent with the Macrophage's Infiltration

**Figure 8:**
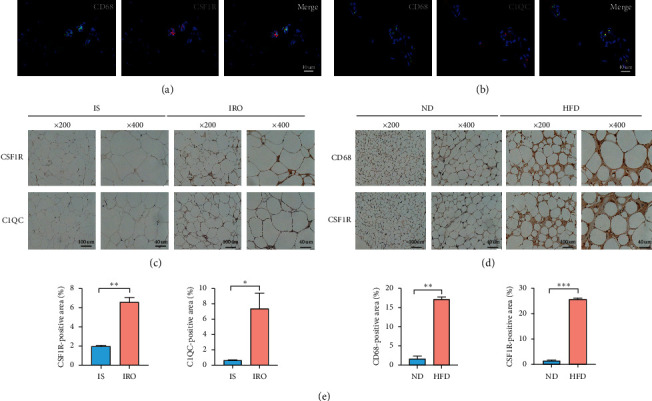
Hub genes' expression was related to macrophage's proinflammation in adipose tissue. (a) Immunofluorescence staining for *CD68* and *CSF1R* in the VAT tissue from IRO patients. Scale bar = 40 um. (b) Immunofluorescence staining for *CD68* and *C1QC* in the VAT tissue from IRO patients. Scale bar = 40um. (c) Immunohistochemical staining for *CSF1R* and *C1QC* in the VAT tissue between IRO patients group and IS group. The scale bar is 100 um and 40 um, respectively. (d) Immunohistochemical staining for *CD68* and *CSF1R* in the VAT tissue between ND-fed mice group and HFD-fed mice group. (e) Quantitative analysis of IHC results of hub genes. ^*∗*^*P* value <0.05. ^*∗∗*^*P* value <0.01. ^*∗∗∗*^*P* value <0.001.

Given that the results of immune cell infiltration analysis showed that the macrophage's infiltrations and functions were associated with the expression of the hub genes, we performed IF staining on the VAT tissue from IRO patients and examined the colocalization relationship between the hub genes and the adipose tissue macrophage marker (CD68). We found the colocalization of *CSF1R* or *C1QC* with *CD68* in adipose tissue (Figures 8(a) and 8(b)). The IHC staining results showed that the increased expression of the hub genes was consistent with more macrophage's infiltration in the VAT tissue from HFD-induced obese mice or from ISO patients versus those in the VAT tissue from ND-fed mice and human control group (Figures 8(c)–8(e)).

## 4. Discussions

Our study's primary goal was to explore significant genes associated with immune cells infiltration in the chronic inflammatory adipose tissue under IR condition. Here, three most significant hub genes (*CSF1R*, *C1QC*, and *TYROBP*) were found and verified by performing RT q-PCR analysis. Meanwhile, the relationship between macrophages infiltration and hub genes' upregulated expression was verified by IHC and IF analysis in mice and human.

The results of GO analysis and KEGG pathway analysis showed that DEGs mainly participated in multiple immune cells causing chronic inflammation reaction in adipose tissue. The GSEA analysis results revealed that high expression of *CSF1R* correlated with TNFR2 noncanonical NF-*κ*B pathway and Type 1 diabetic pathways activated, while the mTOR signaling pathway was inhibited, which participated in the insulin-sensitivity regulation [30]. Meanwhile, the datasets which highly expressed *C1QC* or *TYROBP* mainly correlated with chemokine and JAK-STAT or TLR4 cascade pathway activation, respectively, which participated in diverse immune cells' functional process: the chemokine can induce macrophage polarization under inflammation stage, while JAK-STAT pathway are crucial in NK-Cells' proinflammation, and TLR4 was proved to mediate CD8^+^ T cells' activation in some innate immune disease [31–33]. Then, we verified the hub genes' upregulation was associated with chronic inflammation induced IR or diabetes in SAT or VAT from HFD-induced obese mice and obese patients with T2DM. Furthermore, the downregulated hub genes in AT along with patients' IS improving after diet control and exercise in GSE35411 and our q-PCR analysis results suggested that hub genes' upregulation in AT was accompanied with the process of obtained obesity induced IR. Of note, the q-PCR results showed that three hub genes were upregulated in both SAT and VAT, more obviously in VAT from mice or human, which suggested that the three hub genes seemed more strongly correlated with VAT chronic inflammation and was consistent with former research found that inflammation of VAT played a greater role in obese-induced IR than that of SAT [34].

Considering former results of KEGG and GO analysis that pathways were related to multiple immune cells reactive pathways, we performed immune cells infiltration analysis. The results showed that T cells follicular helper and Macrophage M0 in the IRO group were presented at significant higher fractions compared with IS group. T cells follicular helper had been proved to be related to auto-immune disorders in several immune diseases [35]. M0 macrophage, also called naïve macrophage, came from blood monocyte's recruitment and resident induced by local tissue's proinflammatory microenvironment and also proved to play a crucial role in tissue inflammation process [36]. As mentioned before, the GSEA results showed that the up-regulation of three hub genes were related to multiple immune cells functional pathway activation, so we performed the correlation analysis between infiltrated immune cells and hub genes. Considering three hub genes' upregulation were more obvious in visceral adipose tissue based on our q-PCR analysis, we chose dataset GSE84599 including subcutaneous and visceral adipose tissues from patients with obesity. The results showed that *TYROBP* and M0 macrophages, *CSF1R* and M0 macrophages, plasma cells, or CD8 T cells had positive correlations. M0 macrophages and plasma cells had been proved to promote IR in obese adipose tissue [37]. While CD8 T cells were considered as contributors of macrophage recruitment or activation, which induced adipose chronic inflammation and had a positive correlation with screened hub genes, and there was no different characteristic of infiltration in IRO compared with the IS group, which indicated its limited function within our study. So, we focused on the M0 macrophages infiltrated with highest fraction in AT of IRO compared with that of IS, which also shows positive correlation with hub genes in our study [38]. Furthermore, previous researches illustrated that macrophage played a major function in inflammatory adipose tissue [39]. Thus, we tried to explore the relationship between macrophage infiltration and hub genes' protein in adipose tissue by colocalization measurements, IHC and IF analysis. Of note, the results verified our bioinformatic analysis predicted outcome. Macrophages recruitment from monocytes was a critical step of the production of adipose tissue inflammation; several studies revealed a role of adipose tissue macrophage proliferation in the early stages of obesity and in sustaining adipose tissue inflammation [40, 41]. *CSF1R* and its ligands *M-CSF* and *IL34* stimulated the differentiation and survival of macrophages in local tissues synergistically, which played a crucial role in the process of tissue macrophage's proinflammation functions [42, 43]. Another hub gene *TYROBP* had a function of facilitating the ability of *CSF1R*, while *C1QC* was a C1q/TNF-related protein, which also played a role in innate immune induced auto-immune disease [44]. Numerous studies have reported these three hub genes are related to these immune cells' infiltration, especially macrophages' in the chronic inflammation or dysfunction of adipose, thus contributing to the IR situation happening and serving as a promoter of diabetic pathogenesis. Furthermore, these results were verified based on human specimen or animal models, which indicated that three hub genes' upregulation in AT was associated with obtained obesity induced IR. The present study provided a strong evidence of the significance of three hub genes in AT inflammation or dysfunction and also provided us with a new direction to study their functions that integrate obesity-induced autoinflammation and IR. However, the study is limited by the lack of the definite mechanism of these three hub genes' action of participating in macrophages' function of proinflammation in dysfunctional AT induced IR, and this should be explored in myeloid cells in further researches.

Based on the results above, we can conclude that the upregulation of three hub genes (*CSF1R*, *TYROBP*, and *C1QC*) are associated with immune cells' infiltration and may regulate their maintenance and differentiation, especially macrophage, in the low-grade inflammation stage of obese adipose tissue and thus promote AT dysfunction induced IR.

## 5. Conclusions

Through comprehensive bioinformatics analyzing and experimental verification, our study found that three hub genes *CSF1R*, *C1QC*, and *TYROBP* were associated with immune cells infiltration, especially macrophages in adipose tissue under obesity induced IR condition. Further research on the function of immune cells related to these three genes in obesity-induced IR will help us better understand the mechanism of IR.

## Data Availability

The data used to support this study will be made available from the corresponding author upon request (1931136@tongji.edu.cn).

## Supplementary Materials

Supplementary Materials Supplemental Table 1 includes basic characteristics of type 2 diabetic obese patients and controls. Supplemental Table 2 mainly describes the sequences of primers used in q-PCR. Supplemental Table 3 demonstrates adjust *P* value and logFC of screened 30 DEGs. Supplemental Table 4 shows information of three degrees of 15 top common expressed DEGs. Supplemental Table 5 describes Module A's node genes' related measured index. And, Supplemental Figure 1 presents downregulation of hub genes in IS improving weight-maintenance phase in GSE35411. Supplemental Table 1: basic characteristics of type 2 diabetic obese patients and controls (mean ± SD). FPG: fasting plasma glucose, data represent mean ± SD. ^*∗∗*^*P* value < 0.01 vs. control group. Supplemental Table 2: summary of primer sequences used for RT-qPCR. Supplemental Table 3: 30 commonly expressed DEGs screened with the criterion of adjust *P* value < 0.05. Supplemental Table 4: the 15 top common expressed DEGs across three datasets measured by three degrees in Cytoscape. Supplemental Table 5: node genes measured in three indexes of genes in module A. Supplemental Figure 1: downregulation of hub genes in IS improving weight-maintenance phase in GSE35411 (^*∗*^*P* value < 0.05).
