# Supplementary material for: Identification of Three Significant Genes Associated with Immune Cells Infiltration in Dysfunctional Adipose Tissue-Induced Insulin-Resistance of Obese Patients via Comprehensive Bioinformatics Analysis
Source: Int J Endocrinol. 2021 Jan 22;2021:8820089. doi: 10.1155/2021/8820089 (PMC7850849; doi:10.1155/2021/8820089)

Supplemental Table 1. Basic characteristics of type 2 diabetic obese patients and controls (mean±SD)

	Age	Gender (M/F)	Weight (kg)	Height (cm)	BMI (kg/m^2)	FPG (mmol/L)	HbA1C (%)
**IS (n=5)**	56±6.51	2/3	62.5±6.59	160.8±9.1	24.18±1.55	5.22±0.82	4.96±0.12
**IRO (n=5)**	34.4±11.31	3/2	110.34±22.21	165.6±5.3	40.24±8.18**	7.02±1.47	6.8±0.86**

FPG: fasting plasma glucose, Data represent mean ±SD. **P value<0.01 vs. control group.

Supplemental Table 2. Summary of primer sequences used for RT-qPCR

Gene (Mouse)	Sequence	
**36B4**	Forward:	5' AAG CGC GTC CTG GCA TTG TCT3'
	Reverse:	5' CCG CAG GGG CAG CAG TGG T 3'
**TYROBP**	Forward:	5' CCC AAG ATG CGA CTG TTC TTC 3'
	Reverse:	5' GTC CCT TGA CCT CGG GAG A 3'
**CSF1R**	Forward:	5' GAC CCT GAA TCT CCC GGA AG 3'
	Reverse:	5' GGT ACA ACG GTA GGT CCC AG 3'
**C1QC**	Forward:	5' GGA CGG GCA TGA TGG ACT C 3'
	Reverse:	5' TTC TGT TTG TAT CGG CCC TCC 3'

Gene (human)	Sequence	
**18srRNA**	Forward:	5' GTA ACC CGT TGA ACC CCA TT 3'
	Reverse:	5' CCA TCC AAT CGG TAG TAG CG 3'
**TYROBP**	Forward:	5' ACT GAG ACC GAG TCG CCT TAT 3'
	Reverse:	5' ATA CGG CCT CTG TGT GTT GAG 3'
**CSF1R**	Forward:	5' GGG AAT CCC AGT GAT AGA GCC 3'
	Reverse:	5' TTG GAA GGT AGC GTT GTT GGT 3'
**C1QC**	Forward:	5' AGG ATG GGT ACG ACG GAC TG 3'
	Reverse:	5' GTA AGC CGG GTT CTC CCT TC 3'

Supplemental Table 3: **30 commonly expressed DEGs screened with the criterion of adjust P value < 0.05.**

Dataset	GSE26637		GSE55200		GSE27951	
**item**	adj.P.Val	logFC	adj.P.Val	logFC	adj.P.Val	logFC
**AGPAT9**	0.04519139	-1.9928709	0.00240209	-1.7607205	0.0078735	-2.59138
**AZGP1**	0.00472905	-2.6933204	0.000057	-1.2010294	0.0191697	-1.5627873
**C1QC**	0.00704368	1.68620184	0.00105074	1.30892911	0.00915404	1.34165792
**CD14**	0.02903238	1.4717209	0.00018284	1.2502385	0.00869382	1.60174306
**CD163**	0.0423012	1.55662533	0.00025533	1.92441829	0.00439782	1.5551218
**CHI3L1**	0.01728097	1.35174399	0.00175412	2.38142797	0.01040376	1.28393556
**CPA3**	0.00470132	2.8103774	0.00988517	1.09096226	0.0234259	1.24048682

**CSF1R**	0.04967523	1.22549938	0.00186747	1.06269301	0.00566927	1.19298158
**CTSS**	0.00868886	2.21366745	0.00073858	1.95602201	0.01062991	1.13945304
**EGFL6**	0.01797568	5.94188898	0.0000335	4.25984323	0.00135512	5.76999774
**F13A1**	0.00731403	1.92333973	0.00105466	1.58685523	0.00566927	1.94490756
**GLIPR1**	0.00806924	1.82056229	0.00011768	1.81202707	0.02374759	1.0217565
**GPR137B**	0.00039253	2.59188921	0.00063175	1.31125336	0.00642285	1.10709188
**GPR183**	0.0094948	1.99241317	0.00030686	1.59054317	0.01093567	1.1326671
**HPGDS**	0.02304208	1.65643333	0.0075545	1.39060375	0.00419154	1.11175674
**LYZ**	0.00256153	2.07844913	0.0000335	2.33646229	0.01975614	1.20754834
**MMP9**	0.00425476	3.60589158	0.00045118	2.78054382	0.0191697	1.04911908
**MPEG1**	0.01102319	2.09934075	0.00108424	1.28821919	0.00391054	1.601917
**MRC1**	0.04861885	1.6869311	0.00105245	1.4993624	0.00363163	1.78570942
**MS4A4A**	0.00459704	2.43189743	0.01654572	1.26916719	0.00536005	1.5622467
**MS4A6A**	0.00980783	1.68148543	0.00152616	1.35482459	0.00306827	1.27517444
**NCF4**	0.01890602	1.10276741	0.00069763	1.0431129	0.00364892	1.27080062
**RASSF2**	0.01042758	1.89963848	0.0000366	1.45417735	0.02702299	1.04251308
**RNASE6**	0.00301195	1.70196547	0.00272933	1.06711815	0.00364892	1.5035469
**SFRP4**	0.01697967	2.98620636	0.00066859	2.08400676	0.01040376	1.69665736
**SIGLEC1**	0.00961541	1.15186016	0.00062177	1.14549075	0.00364892	1.1222956
**SLIT2**	0.00384933	1.2495788	0.00000363	1.41701056	0.00569882	1.03757892
**TNMD**	0.00473808	2.16296092	0.00077847	1.82611576	0.02066304	1.30645022
**TYROBP**	0.00425476	2.25225615	0.00025359	1.96352148	0.01040376	1.52437288
**VSIG4**	0.01342969	2.50974512	0.00037849	1.77386698	0.00442355	1.70864357

Supplemental Table 4: The 15 top common expressed DEGs across three datasets measured by three degrees in Cytoscape.

Rank	Name	Degree Score	Closeness Score	MNC Score
**1**	TYROBP	17	17.5	17
**2**	CSF1R	16	15.5	13
**2**	C1QC	16	15	12
**4**	CD163	11	15	12
**5**	MS4A6A	10	14	10
**5**	VSIG4	10	14	10
**7**	CD14	9	13.5	9
**7**	MRC1	9	13.5	9
**9**	F13A1	8	13	8
**9**	MS4A4A	8	13	8
**9**	SIGLEC1	8	13	8
**12**	MMP9	7	12.5	7
**12**	LYZ	7	12.5	7
**12**	CTSS	7	12.5	7
**15**	RNASE6	5	11.5	5

Supplemental Table 5 Node genes measured in three indexes of genes in module A

Gene	Betweenness	Closeness	Degree
**CSF1R**	3.22857	9.5	12
**TYROBP**	2.37619	10	10
**C1QC**	2.37619	10	10
**VSIG4**	1.79048	9.5	10
**CD14**	1.55714	9.5	9
**MRC1**	1.30476	9	9
**F13A1**	0.98571	9	8
**SIGLEC1**	0.98571	9	8
**MS4A6A**	1.01905	8.5	8
**CD163**	1.10476	8.5	7
**MS4A4A**	1.27143	8.5	7

Supplemental Figure 1 :Downregulation of hub genes in IS improving weight-maintenance phase in GSE35411(*P value< 0.05).

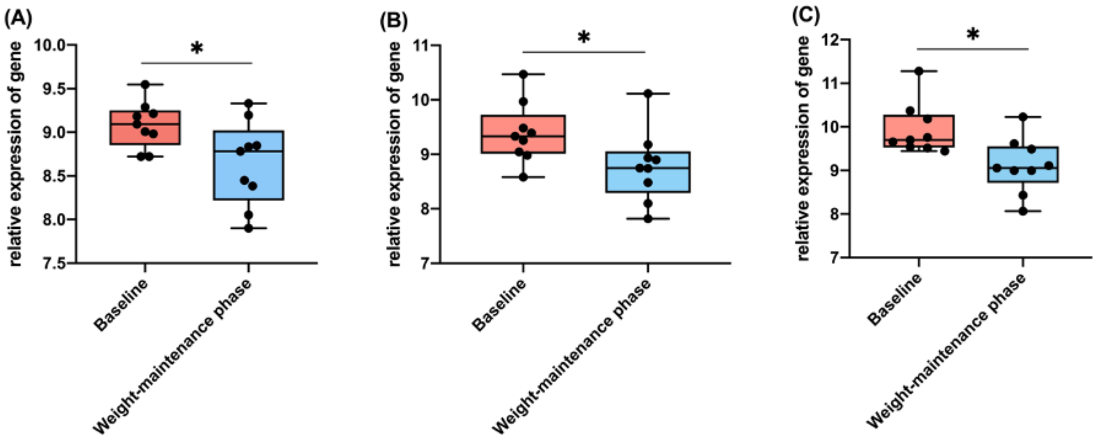

Supplement: Supplementary Materials — Supplemental Table 1 includes basic characteristics of type 2 diabetic obese patients and controls. Supplemental Table 2 mainly describes the sequences of primers used in q-PCR. Supplemental Table 3 demonstrates adjust P value and logFC of screened 30 DEGs. Supplemental Table 4 shows information of three degrees of 15 top common expressed DEGs. Supplemental Table 5 describes Module A's node genes' related measured index. And, Supplemental Figure 1 presents downregulation of hub genes in IS improving weight-maintenance phase in GSE35411. Supplemental Table 1: basic characteristics of type 2 diabetic obese patients and controls (mean ± SD). FPG: fasting plasma glucose, data represent mean ± SD. ∗∗P value < 0.01 vs. control group. Supplemental Table 2: summary of primer sequences used for RT-qPCR. Supplemental Table 3: 30 commonly expressed DEGs screened with the criterion of adjust P value < 0.05. Supplemental Table 4: the 15 top common expressed DEGs across three datasets measured by three degrees in Cytoscape. Supplemental Table 5: node genes measured in three indexes of genes in module A. Supplemental Figure 1: downregulation of hub genes in IS improving weight-maintenance phase in GSE35411 (∗P value < 0.05). [file 8820089.f1.pdf]
